# Controlled Bidirectional Quantum Secure Direct Communication

**DOI:** 10.1155/2014/694798

**Published:** 2014-05-05

**Authors:** Yao-Hsin Chou, Yu-Ting Lin, Guo-Jyun Zeng, Fang-Jhu Lin, Chi-Yuan Chen

**Affiliations:** ^1^Department of Computer Science and Information Engineering, National Chi Nan University, No. 1, University Road, Puli, Nantao 545, Taiwan; ^2^Department of Computer Science and Information Engineering, National Ilan University, No. 1, Section 1, Shen-Lung Road, I-Lan 260, Taiwan

## Abstract

We propose a novel protocol for controlled bidirectional quantum secure communication based on a *nonlocal swap* gate scheme. Our proposed protocol would be applied to a system in which a controller (supervisor/Charlie) controls the bidirectional communication with quantum information or secret messages between legitimate users (Alice and Bob). In this system, the legitimate users must obtain permission from the controller in order to exchange their respective quantum information or secret messages simultaneously; the controller is unable to obtain any quantum information or secret messages from the decoding process. Moreover, the presence of the controller also avoids the problem of one legitimate user receiving the quantum information or secret message before the other, and then refusing to help the other user decode the quantum information or secret message. Our proposed protocol is aimed at protecting against external and participant attacks on such a system, and the cost of transmitting quantum bits using our protocol is less than that achieved in other studies. Based on the *nonlocal swap* gate scheme, the legitimate users exchange their quantum information or secret messages without transmission in a public channel, thus protecting against eavesdroppers stealing the secret messages.

## 1. Introduction

There have been many ingenious applications of quantum information science through the combination of quantum communication and quantum cryptography [1, 2] since Bennett and Brassard  [3] first proposed the original quantum key distribution (QKD) protocol in 1984, which is a way for two remote users to share a private key for encrypting or decrypting secret messages in a quantum channel. This was one of the most promising applications of quantum machine, and many more QKD protocols have since been presented [4–8].

The quantum secure direct communication (QSDC) protocol differs from the QKD protocols used to distribute private keys and has been proposed [9] for directly transmitting secret messages, without having to share a private key between two legitimate users beforehand. Moreover, Boström and Felbinger [10] presented a “Ping Pong” QSDC using Einstein-Podolsky-Rosen (EPR), but some researches [11–13] noted that the “Ping Pong” protocol is insecure for direct communication in a noisy quantum channel. Deng et al. [14] also presented a two-step QSDC protocol using EPR pairs, which is useful for QSDC protocols not sharing the private key first; quantum bits (qubits) carrying the secret messages are transmitted directly. Therefore, bidirectional QSDC (BQSDC) is a concept extended from QSDC protocols. Most QSDC protocols offer only one way communication, so that the secret message can only be transmitted from one legitimate user to the other. If two remote legitimate users want to exchange their respective secret messages using the QSDC protocol, they have to implement it twice. In this situation, one legitimate user can receive the secret message from the other, but fail to keep their promise to transmit their own message. Thus, BQSDC protocols must be designed in such a way that two remote legitimate users transmit their respective secret messages simultaneously in one way communication. Nguyen [15] improved the ping pong protocol, and first proposed the BQSDC protocol (called quantum dialogue protocol) which enables two remote legitimate users to exchange secret messages. Other BQSDC protocols [15–17] are based on the QSDC protocols. Legitimate users must transmit the qubit with the secret message in the public channel under any local operation and classical communication (LOCC) in order to obtain the secret message from the other party; however, an eavesdropper could steal the qubits or attack the protocol without being discovered. To prevent an external eavesdropper extracting the secret messages, researchers developed BQSDC protocols that do not transmit encoded qubits [15]. In general, BQSDC protocols assume that participants are honest, so they are unable to protect against participant attacks by dishonest participants utilizing the order of measurement announce.

**Figure 1:**
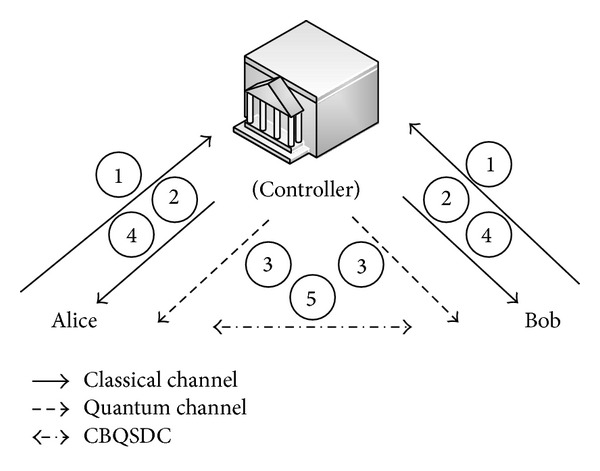
The demonstration of online shopping.

To prevent this asymmetric situation, we suggest that a fair third party should be involved to authenticate participants and prevent the above situation. In most one-way or bidirectional protocols, third parties are designed to identify the participants, so receivers must get permission from the third party to obtain the secret messages. In our proposed protocol, we call the fair third party the controller or supervisor (represented as Charlie). This controller not only provides authentication of legitimate users, but also prevents participant attacks in the QSDC protocol. In some applications, we need a powerful third-party to assist the process or provide costly equipment [18, 19]. We take a simple example of online shopping to explain why controller is needed [see Figure 1]. Assume the controller is the online shopping mall, Alice and Bob are users and the detailed steps are described as follows.

 Step 1: Alice and Bob send registration request to the controller.
 Step 2: controller authenticates them as members.
 Step 3: controller transmits GHZ sequences to Alice and Bob.
 Step 4: they check the channel security with classical bits transmitted.
 Step 5: Alice and Bob exchange their quantum information with our protocol.

Most QSDC protocols claim that their protocol can safely transmit secret messages by qubits via a public channel; however, eavesdroppers can still steal or attack the qubits in transmission. Some researchers have taken advantage of entanglement swapping to design QSDC protocols that exclude the encoded qubit transmission process. Yan and Zhang [20] presented a scheme for QSDC based on teleportation without transmitting a qubit with a secret message. Using the teleportation scheme, the legitimate user can send an unknown quantum state through a quantum channel to another user. Before Yan's protocol, there were many QSDC protocols that just transmitted classical information instead of quantum information.

Due to the quantum property of noncloning [21], quantum information must be transmitted from the sender to the receiver using entanglement swapping [22]. In addition, it is far more difficult to produce quantum resources than classical ones. If we use quantum resources to send classical messages, we may sometimes find that the cost of the quantum resources is higher than that of the secret message itself. Overall, if the QSDC protocol can transmit quantum information, it is also able to transmit classical messages, but not vice versa. So it takes more effort to come up with a QSDC protocol that transmits quantum information than one that transmits classical information.

Therefore, we propose a novel protocol for controlled bidirectional QSDC based on a* nonlocal swap* gate scheme without transmitting the qubits carrying the secret message. Legitimate users can simultaneously exchange their respective quantum information or classical messages with each other, with the controller's permission. Our protocol has the ability to transmit quantum information, which is rare in QSDC protocols. This is advantageous because when we use quantum resources to transmit classical messages, sometimes the cost will be higher than the resource cost in using classical cryptography, which can achieve the same goal. Moreover, quantum information is noncloning. This means that an arbitrary quantum state cannot be reproduced if we do not know its actual state; this makes quantum information more secure than classical information. We prove that our scheme is reliable by analyzing the security; the analysis shows that our protocol can resist both internal and external attacks. Moreover, we ensure that it is impossible for one participant to quickly receive the other's message. Performance comparison is also provided, and our quantum resource costs are shown to be the lowest. [23] demonstrates that a* nonlocal swap* gate requires at least two EPR pairs. Our protocol uses 5 qubits to accomplish communication, and the supernumerary one qubit is used for the controller. Compared to other CQSDC protocols, the cost of our proposed protocol is the lowest.

In Section 2, we present works related to our protocol. In Section 3, we present the controlled bidirectional QSDC protocol based on the* nonlocal swap* gate. In Section 4, we analyze the security of our protocol. In Section 5, we compare the performance of our protocol with previous QSDC protocols. Finally, Section 6 offers conclusions drawn from this paper.

## 2. The Nonlocal SWAP Gate Scheme

The swap gate plays an important role in network design for qubit quantum computation. The quantum operation of the local swap gate [24, 25] permutes the state of two qubits; therefore, we propose that legitimate users can interchange their information with a swap gate as follows:

(1) $$\begin{array}{c} U_{\text{swap}}{|\psi \rangle }_{1}{|\phi \rangle }_{2}={|\phi \rangle }_{1}{|\psi \rangle }_{2}. \end{array}$$

It can be represented by the following matrix:

(2) $$\begin{array}{c} U_{\text{swap}}=\left[\begin{array}{cccc} 1 & 0 & 0 & 0\\ 0 & 0 & 1 & 0\\ 0 & 1 & 0 & 0\\ 0 & 0 & 0 & 1 \end{array}\right]. \end{array}$$

**Figure 2:**
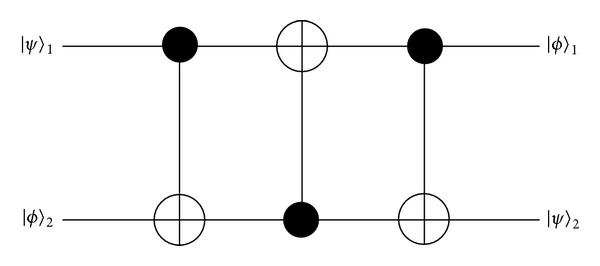
The* swap* gate cascades three quantum* Controlled-Not* gates.

On the quantum circuit, this can be achieved by cascading three quantum* Controlled-NOT* (CNOT) gates [see Figure 2] for arbitrary qubit states |*ψ*〉_1_ and |*ϕ*〉_2_ as follows:

(3) $$\begin{array}{c} C_{12}C_{21}C_{12}{|\psi \rangle }_{1}{|\phi \rangle }_{2}={|\phi \rangle }_{1}{|\psi \rangle }_{2}. \end{array}$$

We define *C*_*ij*_ as a notation of a quantum CNOT gate. The first qubit *i* is a control bit, which performs the* NOT* operation on the second target qubit *j* only when the control qubit *i* is |1〉 as follows:

(4) $$\begin{array}{c} |\psi_{1}\rangle |\psi_{2}\rangle \overset{C_{12}}{\to }|\psi_{1}\rangle |\psi_{1}⊕\psi_{2}\rangle \\ |\psi_{1}\rangle |\psi_{2}\rangle \overset{C_{12}}{\to }|\psi_{1}⊕\psi_{2}\rangle |\psi_{2}\rangle , \end{array}$$

where ⊕ denotes addition modulo 2.

Because the framework of the bidirectional QSDC protocols is established on two remote legitimate users who want to exchange secret messages, we have to use the swap gate in a nonlocal manner. Fortunately, Barenco et al. [26] proposed a* nonlocal swap* gate scheme that can be used to construct a bidirectional QSDC protocol. We will introduce this* nonlocal swap* gate scheme below.

**Figure 3:**
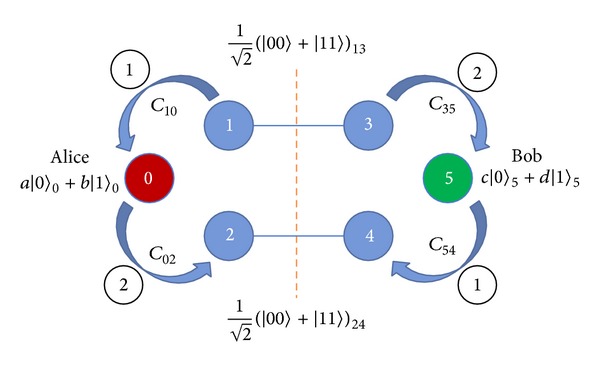
The demonstration of* nonlocal swap* gate scheme.

Suppose that two remote legitimate users, Alice and Bob, want to swap their respective unknown qubits |*ψ*〉_0_ = *a*|0〉 + *b*|1〉 and |*ψ*〉_5_ = *c*|0〉 + *d*|1〉 with each other. To accomplish this task, they have to share two quantum pairs previously with the same maximally entangled state |*ϕ*〉_13_ = (1/2)(|00〉 + |11〉) and |*ϕ*〉_24_ = (1/2)(|00〉 + |11〉). Therefore, there are three qubits 0, 1, and 2 given by Alice, and another qubits 3, 4, and 5 given by Bob. To interchange qubit 0 and qubit 5, Alice and Bob will perform the following protocol [see Figure 3].

**Step 1**

Alice implements *C*_10_ (the CNOT gate on qubit 1 and qubit 0) and then *C*_02_ while Bob performs *C*_54_ and then *C*_35_.

**Step 2**

After Alice measures her qubit 2 and Bob measures his qubit 4, they communicate the result to each other. If the results are the same, they go to Step 3, or Alice and Bob apply the* NOT* gate to the remaining qubits in their possession. The* NOT* gate can be presented by the following matrix:

(5) $$\begin{array}{c} \left[\begin{array}{cc} 0 & 1\\ 1 & 0 \end{array}\right]. \end{array}$$

**Step 3**

Alice and Bob apply the rotation to qubit 1 and qubit 3, respectively. Consider the following:

(6) $$\begin{array}{c} \sqrt{\frac{1}{2}}\left[\begin{array}{cc} 1 & 1\\ 1 & -1 \end{array}\right]. \end{array}$$

**Step 4**

Alice measures her qubit 1 and Bob measures his qubit 3; they then communicate the result to each other. If the results are the same, the qubit state will have been swapped. Otherwise, Alice and Bob apply the unitary transformation

(7) $$\begin{array}{c} \left[\begin{array}{cc} 1 & 0\\ 0 & -1 \end{array}\right] \end{array}$$

to qubit 0 and qubit 5, respectively, with the disagreeing results. Finally, they successfully swap their quantum information to a different place.

This protocol not only successfully swaps quantum information to different places, but also simultaneously exchanges the quantum information. It is suitable for bidirectional QSDC protocol, but it cannot protect against one legitimate user deriving the quantum information from the other side first, and then not assisting the other side in decoding their quantum information. Therefore, we designed a new protocol with a controller in order to avoid an uncoordinated condition between the legitimate users based on Barenco's protocol.

## 3. Controlled Bidirectional Quantum Secure Direct Communication

Before introducing our protocol for controlled bidirectional QSDC based on a* nonlocal swap* gate [26], we need to define four Bell states and three-particle GHZ states in our protocol. The four Bell states are

(8) $$\begin{array}{c} |\Phi^{+}\rangle =\frac{1}{\sqrt{2}}\left(|00\rangle +|11\rangle \right)=\frac{1}{\sqrt{2}}\left(|++\rangle +|--\rangle \right)\\ |\Phi^{-}\rangle =\frac{1}{\sqrt{2}}\left(|00\rangle -|11\rangle \right)=\frac{1}{\sqrt{2}}\left(|+-\rangle +|-+\rangle \right)\\ |\Psi^{+}\rangle =\frac{1}{\sqrt{2}}\left(|01\rangle +|10\rangle \right)=\frac{1}{\sqrt{2}}\left(|++\rangle -|--\rangle \right)\\ |\Psi^{-}\rangle =\frac{1}{\sqrt{2}}\left(|01\rangle -|10\rangle \right)=\frac{1}{\sqrt{2}}\left(|+-\rangle -|-+\rangle \right). \end{array}$$

The eight GHZ states in a three-particle maximally entangled quantum system are as follows:

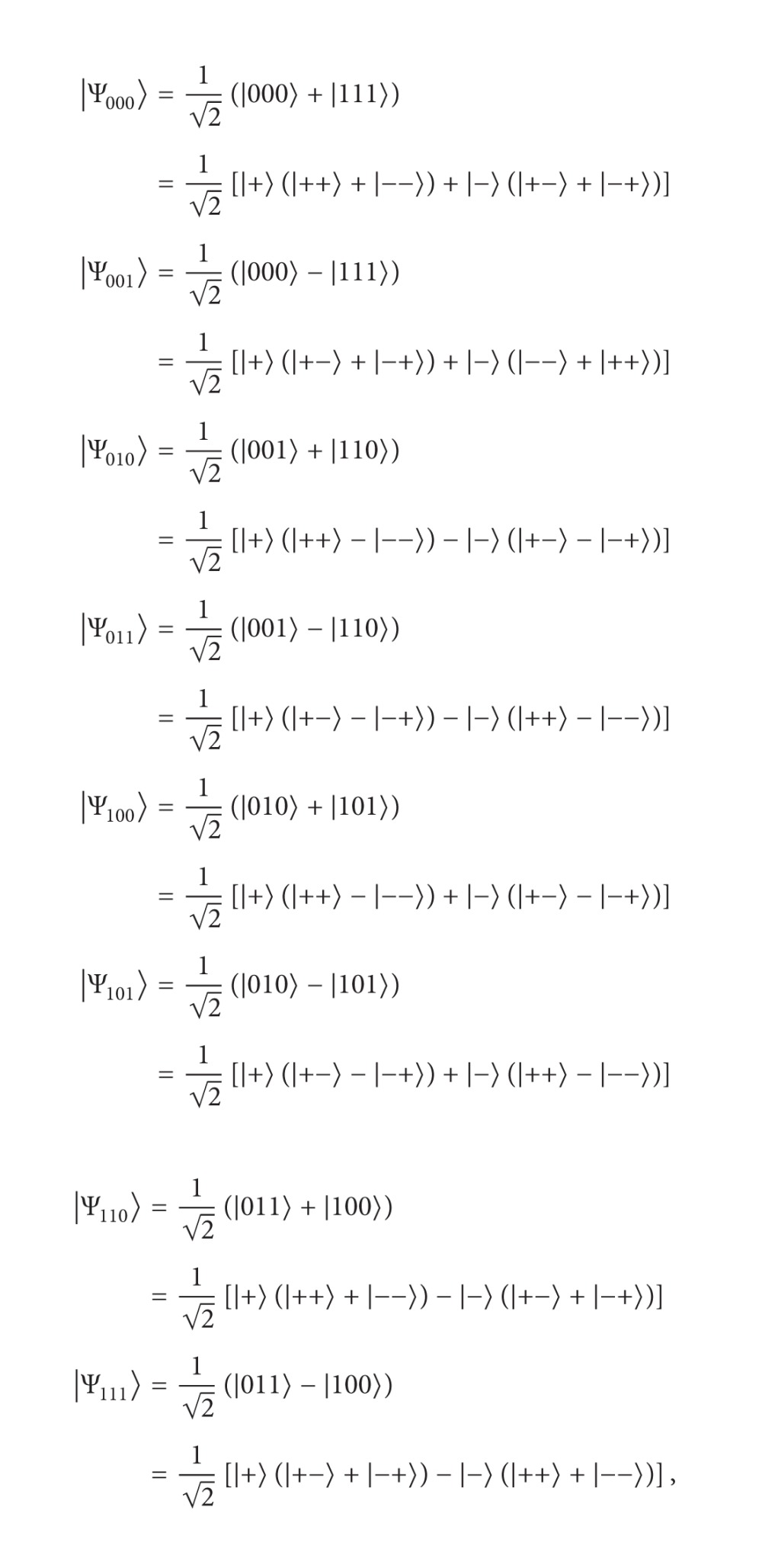
(9)
where $\left|+\rangle =\left(1/\sqrt{2}\right)(|0\rangle +|1\rangle \right)$ and $\left|-\rangle =\left(1/\sqrt{2}\right)(|0\rangle -|1\rangle \right)$. Now, let us describe the CBQSDC protocol. Suppose that the two remote legitimate users, Alice and Bob, want to swap their respective unknown qubit to each other. To accomplish this, they must initially share one GHZ state and one EPR pair. To swap their qubits, Alice and Bob must have permission from Charlie (controller), according to the following protocol.

First, we have to detect whether an eavesdropper exists in the quantum channel and authenticate the legitimate users.

**Step 1**

The controller (supervisor) Charlie generates a group of *N* three-particle GHZ states randomly in one of the eight three-particle GHZ states (|Ψ_*ijk*_〉_*ABC*_, *i*, *j*, *k* = 0, 1) between legitimate users Alice and Bob. For a group of *N* three-particle GHZ states, Charlie keeps the sequence of particles *C* for himself and sends the sequence of particles *A* and the sequence of particles *B* to Alice and Bob, respectively.

**Step 2**

Once Alice and Bob confirm with Charlie that they have received the sequences of particles *A* and *B*, respectively, they have an order to choose the sufficiently random subset of *A* and *B* sequence for detecting an eavesdropper. First, Alice and Bob publish the positions of GHZ states which are used for detection in the quantum channel, and they require that Charlie announce the initial states of the corresponding GHZ states. Once Charlie has published the initial states, Alice and Bob measure the selected particles of sequences *A* and *B* using one of two measuring basis,* Z*-basis |0〉, |1〉 or* X*-basis |+〉, |−〉 randomly, and then announce the measuring bases and results for the selected particles of sequences *A* and *B* through a classical channel. According to the public information, the three parties (Alice, Bob, and Charlie) measure their corresponding particles of *A* sequence, *B* sequence, and *C* sequence using the same bases, respectively, and they will reveal their measurement results for analysis. According to the measurement results of the three parties, they can check whether the quantum channel is secure through the error rate. If the error rate is higher than the predetermined threshold, the communication must be terminated; otherwise, Alice, Bob, and Charlie go to the next step.

**Step 3**

After ensuring the security of the quantum channel, Charlie uses some of the remaining *C* particles to produce EPR pairs between Alice and Bob. Only Charlie measures some of the remaining *C* particles using* X*-basis, and gives the position to Alice and Bob. The particles in the same positions of *A* sequence and *B* sequence will then be maximally entangled with each other between Alice and Bob. Consider the following:

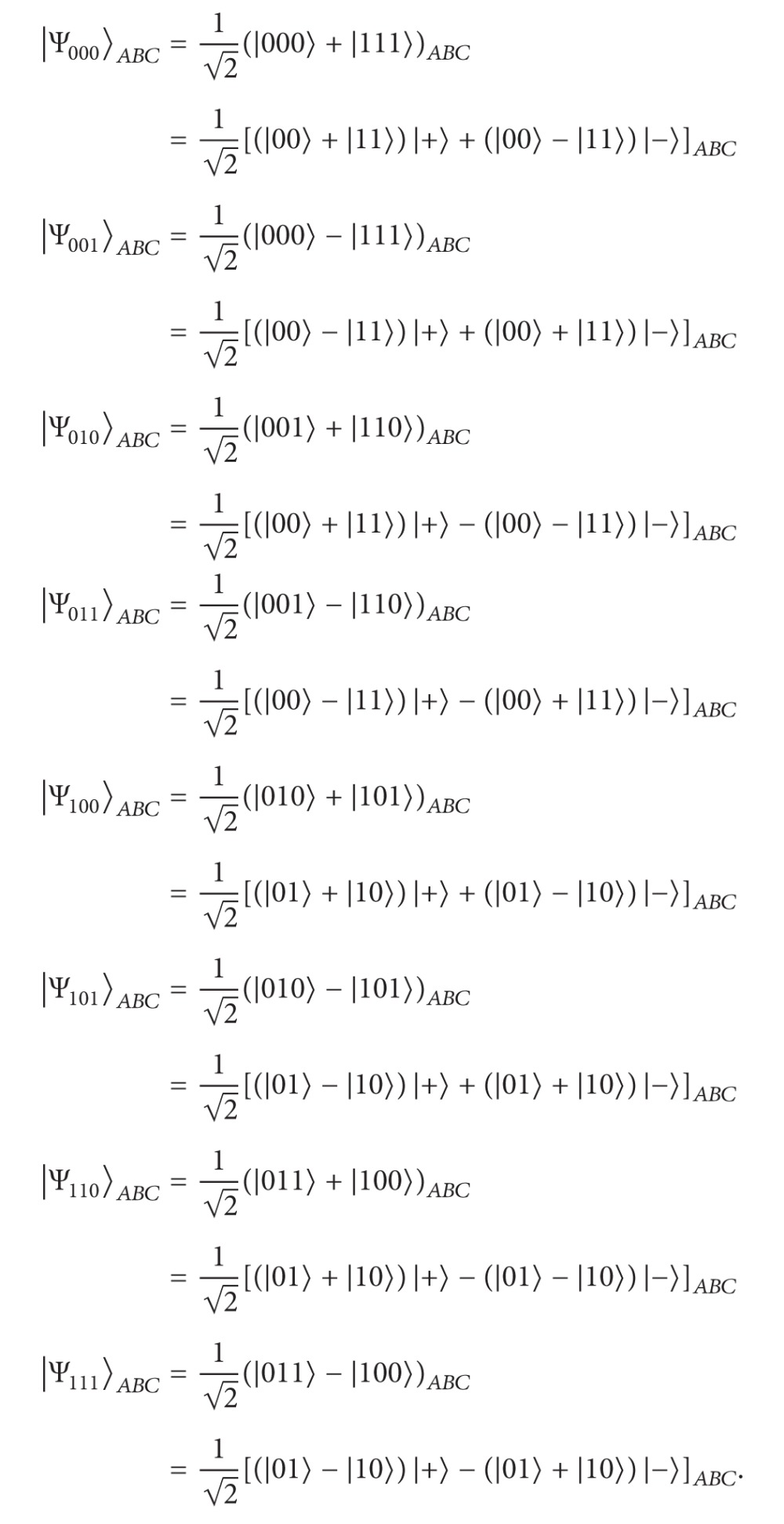
(10)

**Step 4**

**Figure 4:**
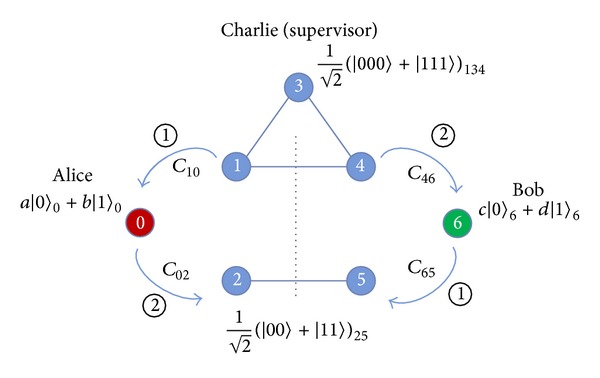
The scenario of our proposed protocol.

After the quantum channel is secure, Charlie prepares EPR pairs and GHZ states from the remaining *C* sequence to implement the protocol. To understand the process of our protocol easily, we will number the qubits [see Figure 4]. Assume that Charlie has already prepared an EPR pair ${|\Phi^{+}\rangle }_{25}=\left(1/\sqrt{2}\right)(|00\rangle +|11\rangle )$ for Alice and Bob, and then a GHZ state ${|\Psi_{000}\rangle }_{134}=\left(1/\sqrt{2}\right)(|000\rangle +|111\rangle )$ for Alice, Charlie, and Bob. Here, the GHZ state and EPR pair can be collocated randomly. Alice and Bob want to swap their respective unknown qubits |*ψ*〉_0_ = *a* | 0〉+*b* | 1〉 and |*ψ*〉_6_ = *c* | 0〉+*d* | 1〉 with each other. Therefore, there are three qubits 0, 1, and 2 given by Alice, and the other three qubits 4, 5, and 6 are given by Bob. The remaining qubit 3 is for controller Charlie [see Figure 4]. The quantum system becomes

(11) |ψ〉0⊗|Ψ000〉134⊗|Φ+〉⊗|ψ〉6 =(a|0〉+b|1〉)0⊗12(|000〉+|111〉)134  ⊗12(|00〉+|11〉)25⊗(c|0〉+d|1〉)6.

**Step 5**

After confirming these steps above, Alice implements *C*_10_ and then *C*_02_ while Bob performs *C*_65_ and then *C*_46_.

**Step 6**

After Alice measures her qubit 2 and Bob measures his qubit 5, they communicate the results to each other and Charlie. If the results are the same, they go to the next step. Otherwise, Alice and Bob apply the* NOT* gate to the remaining qubits in their possession, as in Step 2 of the* nonlocal swap* gate scheme. Suppose that there is a |0〉_2_ ⊗ |1〉_5_ difference between Alice and Bob's measurement results of qubits 2 and 5. Because there are two different results, Alice, Bob, and Charlie have to apply the *X* gate to their remaining qubits 0, 1, 3, 4, and 6. Here, Alice and Bob publish their measurement results of qubit 1 and 4 as |0〉_1_ ⊗ |1〉_4_ and Charlie measures his qubit 3 as |0〉_3_. The measurement result of qubit 3 cannot be published, but according to the results of qubits 1, 2, 4, and 5, Charlie will tell Alice and Bob to apply the *Z* gate to transfer their qubits 0 and 6 to obtain the correct qubit state (*c* | 0〉+*d* | 1〉)_0_ and (*a* | 0〉+*b* | 1〉)_6_. Consider the following:

(12) C46C02C65C10(|ψ〉0⊗|Ψ000〉134⊗|Φ+〉⊗|ψ〉6) =12[|00〉25(ac|00000〉+ad|11110〉         + bd|10001〉+bc|01111〉)01346    +|01〉25(ac|11111〉+ad|00001〉        + bd|01110〉+bc|10000〉)01346    +|10〉25(ac|11111〉+ad|00001〉        + bd|01110〉+bc|10000〉)01346    +|11〉25(ac|00000〉+ad|11110〉        + bd|10001〉+bc|01111〉)01346]

(13) |01〉25(ac|11111〉+ad|00001〉    + bd|01110〉+bc|10000〉)01346 →X01346|01〉25(ac|00000〉+ad|11110〉         + bd|10001〉+bc|01111〉)01346.

**Step 7**

Alice, Bob, and Charlie apply the* Hadamard* gate to qubits 1, 4, and 3, respectively, as Step 3 of the* nonlocal swap* gate scheme.

**Step 8**

Alice and Bob measure their respective qubits 1 and qubit 4, and publish the results for Charlie. Once the results from Alice and Bob are published, Charlie measures his qubit 3 before telling Alice and Bob the unitary operation *I* = |0〉〈0 | +|1〉〈1|, *σ*_*x*_ = |0〉〈1 | +|1〉〈0|, *σ*_*z*_ = |0〉〈0 | −|1〉〈1|, *iσ*_*y*_ = |0〉〈1 | −|1〉〈0| to transfer their qubit 0 and qubit 6, which leads to successful swapping as follows:

(14) $$\begin{array}{c} {|01\rangle }_{25}⊗\frac{1}{2\sqrt{2}}\left[\begin{array}{c} {|000\rangle }_{134}{\left(c|0\rangle +d|1\rangle \right)}_{0}{\left(a|0\rangle +b|1\rangle \right)}_{6}+\\ {|001\rangle }_{134}{\left(c|0\rangle -d|1\rangle \right)}_{0}{\left(a|0\rangle -b|1\rangle \right)}_{6}+\\ {|010\rangle }_{134}{\left(c|0\rangle -d|1\rangle \right)}_{0}{\left(a|0\rangle -b|1\rangle \right)}_{6}+\\ {|011\rangle }_{134}{\left(c|0\rangle +d|1\rangle \right)}_{0}{\left(a|0\rangle +b|1\rangle \right)}_{6}+\\ {|100\rangle }_{134}{\left(c|0\rangle -d|1\rangle \right)}_{0}{\left(a|0\rangle -b|1\rangle \right)}_{6}+\\ {|101\rangle }_{134}{\left(c|0\rangle +d|1\rangle \right)}_{0}{\left(a|0\rangle +b|1\rangle \right)}_{6}+\\ {|110\rangle }_{134}{\left(c|0\rangle +d|1\rangle \right)}_{0}{\left(a|0\rangle +b|1\rangle \right)}_{6}+\\ {|111\rangle }_{134}{\left(c|0\rangle -d|1\rangle \right)}_{0}{\left(a|0\rangle -b|1\rangle \right)}_{6} \end{array}\right]. \end{array}$$

Our protocol not only simultaneously exchanges quantum information $\left\{\alpha |0\rangle +\beta |1\rangle ,\left(1/\sqrt{2}\right)(|0\rangle \pm |1\rangle ),|0\rangle ,|1\rangle \right\}$ but also interchanges classical secret messages 0, 1 for each user. The legitimate users first define that $\left|+\rangle =\left(1/\sqrt{2}\right)(|0\rangle +|1\rangle \right)$ represents classical bit “0,” and $\left|-\rangle =\left(1/\sqrt{2}\right)(|0\rangle -|1\rangle \right)$ represents classical bit “1,” then the legitimate users prepare the qubit states |+〉 and |−〉 as their secret messages to implement all of the above steps. After transferring their respective qubit states, they use the* X*-basis to measure their respective qubit 0 and qubit 6. Finally, they successfully swap the secret messages. Here, Alice and Bob publishes their measurement results of qubits 1 and 4 as |0〉_1_ ⊗ |1〉_4_, and Charlie measures his qubit 3 as |0〉_3_. The measurement result of qubit 3 cannot be published, but according to the results of qubits 1, 2, 4, and 5, Charlie will tell Alice and Bob to apply the *Z* gate to transfer their qubits 0 and 6 to obtain the correct qubit state (*c* | 0〉+*d* | 1〉)_0_ and (*a* | 0〉+*b* | 1〉)_6_. Our protocol can, therefore, simultaneously exchange a combination of quantum information and classical secret messages.

## 4. Security Analysis

Most bidirectional QSDC protocols discuss the security of external attack from an eavesdropper (Eve), but seldom or never discuss the honesty between the legitimate users and the controller. They [15–17, 27] all have to assume that the legitimate users are honest and reliable, and then cooperate to decode the classical secret messages from each other. However, there is a problem involving the honesty of the legitimate users, which arises if one of the legitimate users receives the quantum information or secret message from the other first, and then does not cooperate to help the other decode the quantum information or secret message. Thus, we will analyze the security for external attacks from Eve on the two parties, and internal problems from the legitimate users. Furthermore, there are some attacks that use the imperfect quantum equipment to get illegal secret information, like the Trojan horse attack [28, 29], but when the technology of manufacturing quantum resource becomes more mature, this kind of attacks would be prevented.

*External Attack.* To check the security of the quantum channel, we have to suppose that the eavesdropper intends to steal the quantum information or classical messages via the quantum channel. There are ways for Eve to conduct this kind of attack. We introduce how Eve would attack our protocol, and show that these attacks do not allow Eve access to any information about the secret messages.

**Figure 5:**
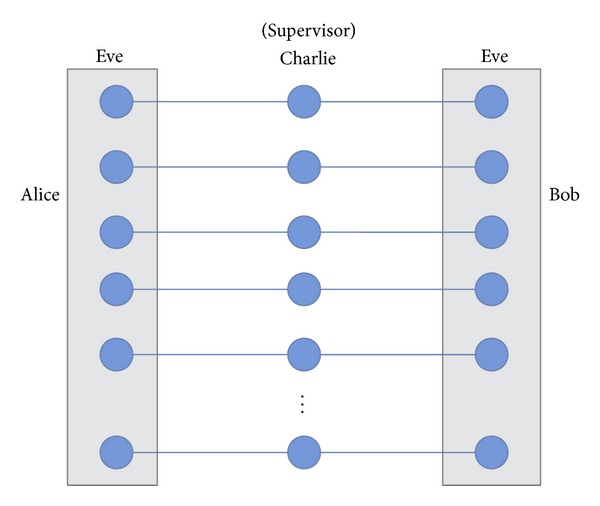
The scenario of Eve intercepts sequences.

**Figure 6:**
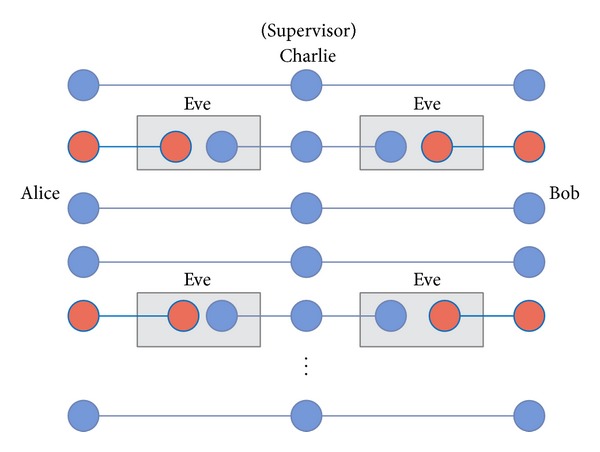
The scenario of Eve inserts EPR pairs.

**Figure 7:**
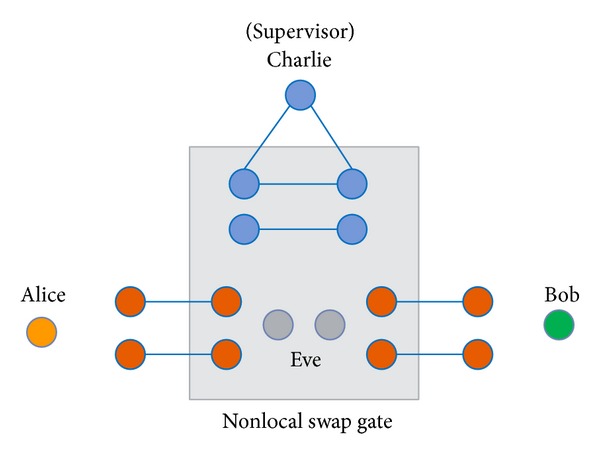
The scenario of Eve steals secret message by the* nonlocal swap* gate scheme.

*(1) The Man in the Middle Attack by Eve.* We suppose that Eve prepares some EPR pairs with the intent to steal secret messages by the* nonlocal swap* gate scheme [see Figure 7]. When Charlie (controller) sends the sequence of *A* particles and *B* particles to Alice and Bob, Eve intercepts the *A* sequence and *B* sequence and keeps some of them [see Figure 5]. Eve then inserts one of the particles of each EPR pair prepared by herself back to the *A* sequence and *B* sequence, and sends *A*′ sequence and *B*′ sequence (*A*′ and *B*′ sequences represent the sequences that contain Eve's EPR pairs.) to Alice and Bob [see Figure 6]. If Eve is not detected and her EPR pairs are the quantum resources for legitimate users to exchange their secret message, she can obtain the secret messages from Alice and Bob. Since the quantum resources are kept between the legitimate users and Eve, Eve can mimic Alice and Bob's actions in order to obtain the secret messages.

**Figure 8:**
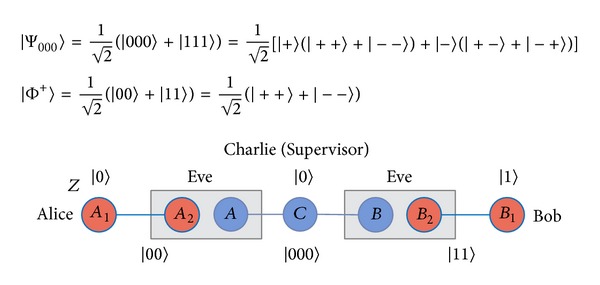
The scenario of external attack in which the legitimate users and controller measure the qubits *A*_1_, *C*, and *B*_1_.

However, Eve would be found out in the quantum channel. The following shows the error detection rate that the controller and legitimate users find an eavesdropper in the quantum channel, and the calculation of Eve's success rate. After Alice and Bob confirm with Charlie that they have received all the sequences of particles *A*′ and *B*′ (*A*′ and *B*′ sequence represent the sequences that contain Eve's EPR pairs), respectively, they have an order to choose the sufficiently random subset of *A* and *B* sequence for detecting an eavesdropper. Assume that Eve inserts the 2*k*_*e*_ EPR pairs (Eve has to use two EPR pairs to replace a GHZ state) [see Figure 8]. The legitimate users now have *k*_*e*_/*N* probability (Charlie prepares a group of *N* three-particle GHZ states) to choose Eve's EPR pairs. If one of the legitimate users chooses the particle that is one of the EPR pairs from Eve for a channel check, the legitimate users have 3/4 probability of finding the error. Assume that Alice chooses the GHZ state $\left(1/\sqrt{2}\right){\left(|000\rangle +|111\rangle \right)}_{ABC}$ that has the two EPR pairs $\left(1/\sqrt{2}\right){\left(|00\rangle +|11\rangle \right)}_{A_{1}A_{2}}$ and $\left(1/\sqrt{2}\right){\left(|00\rangle +|11\rangle \right)}_{B_{1}B_{2}}$ inserted, and Alice keeps the qubit *A*_1_ and Bob keeps the qubit *B*_1_, then Eve keeps the qubits *A*, *B*, *A*_2_, and *B*_2_ [see Figure 8].

If Alice (Bob) measures the qubit *A*_1_ (*B*_1_) using* Z*-basis, the measurement result will have a 1/2 probability of collapsing to |00〉_*A*_1_*A*_2__ (|00〉_*B*_1_*B*_2__) or |11〉_*A*_1_*A*_2__ (|11〉_*B*_1_*B*_2__), and the GHZ state also has a 1/2 probability of collapsing to |000〉_*ABC*_ or |111〉_*ABC*_. When the qubits *A*_1_, *C*, and *B*_1_ are |0〉 or |1〉, Eve has a (1/4)((1/2) × (1/2)) probability of not being found. In other words, if Alice (Bob) measures the qubit *A*_1_ (*B*_1_) using* X*-basis, the measurement result will have a 1/2 probability of collapsing to |++〉_*A*_1_*A*_2__ (|++〉_*B*_1_*B*_2__) or |−−〉_*A*_1_*A*_2__ (|−−〉_*B*_1_*B*_2__), and the GHZ state also has a 1/4 probability of collapsing to |+++〉_*ABC*_, |+−−〉_*ABC*_, |−+−〉_*ABC*_, and |−−+〉_*ABC*_. When the qubits *A*_1_, *C*, and *B*_1_ are |+〉 or |−〉, Eve has a (1/4) ((1/2) × (1/2)) probability of not being found.

The overview of the above two external attacks: Charlie prepares *N* GHZ states for detecting an eavesdropper and quantum resources. Eve prepares 2*k*_*e*_ EPR pairs to insert into the *A* and *B* sequences. The legitimate users randomly choose *m* GHZ states together for detecting quantum channels. Therefore, when the legitimate users choose one of *N* GHZ states, Eve has a ((*k*_*e*_/*N*) × (1/4) + ((*N* − *k*_*e*_)/*N*) × 100%) probability of not being found in the quantum channel. However, if the legitimate users choose *m* number of *N* GHZ states for detecting quantum channels, the error detection rate is 1 − ((*k*_*e*_/*N*) × (1/4) + ((*N* − *k*_*e*_)/*N*) × 100%)^*m*^ for the legitimate users, and the controller finds Eve in the quantum channel regardless of whether the measuring basis is* X*-basis or* Z*-basis.

**Figure 9:**
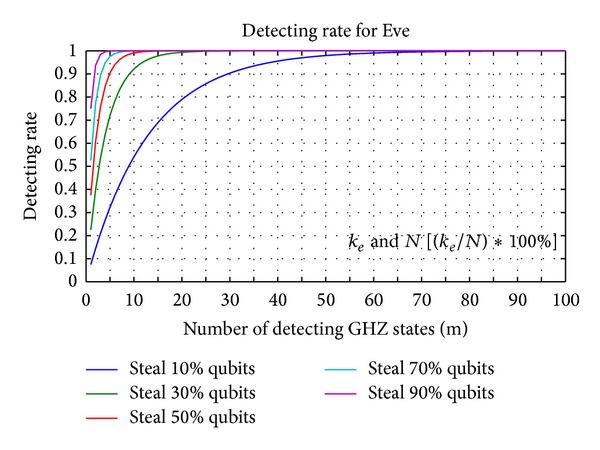
The different percentages of *k*_*e*_ in *N* GHZ states corresponding to the error detection rate and the number of detecting GHZ states. (*k*_*e*_: the number of GHZ states replaced by Eve's EPR pairs; *N*: the number of GHZ states which are prepared from Charlie).

**Figure 10:**
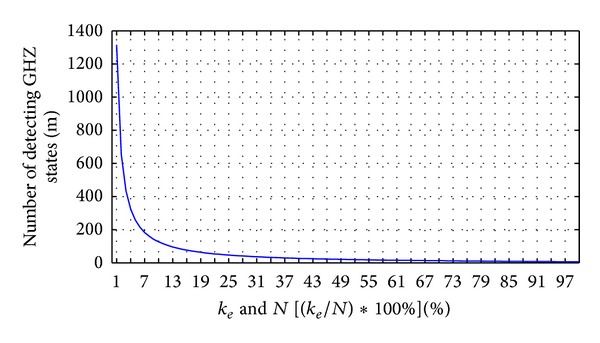
The legitimate users detect eavesdropper with 100% probability corresponding to the number of detecting GHZ states and percentage of replaced GHZ states.

Figures 9 and 10 display the relation between the three parameters *m*, *k*_*e*_, and *N*. In Figure 9, we display five percentages of *k*_*e*_ in *N* GHZ states corresponding to the error detection rate and the number of detecting GHZ states. The legitimate users can depend on the error detection rate to decide how many *m* GHZ states must be used to detect an eavesdropper. For example, suppose that Charlie prepares 100 GHZ states; then, Eve uses 100 EPR pairs to replace 50 GHZ states for the legitimate users. According to the line of 50% *N* in Figure 9, the legitimate users only choose 10 GHZ states for detecting an eavesdropper, the legitimate users and controller find the eavesdropper with a 99.0905% probability. When the legitimate users increase the number of GHZ states for detecting an eavesdropper to 29, there is a 99.9999% probability of the legitimate users and controller finding the eavesdropper. Therefore, the higher the number of GHZ states that are replaced, the fewer detecting GHZ states that are required by the legitimate users to find the eavesdropper.

Figure 10 illustrates that the legitimate users detect the eavesdropper with a 100% probability corresponding to the number of detecting GHZ states and percentage of replaced GHZ states. As in Figure 10, the higher the number of GHZ states that are replaced, the fewer detecting GHZ states that are required by the legitimate users to find the eavesdropper. Conversely, the legitimate users need to consume more detecting GHZ states to detect the quantum channels when Eve replaces fewer GHZ states to be EPR pairs.

*(2) The Teleportation Attack *[30]. Some QSDC protocols examine the security of quantum channel only after photons transmission are all finished, and then this kind of attack will get benefits from this type of transmission. Once photons are transmitted, our proposed protocol will check the error rate to ensure that the quantum channel is secure, so this attack is invalid to our protocol.

*(3) The Correlation-Elicitation *[31–33]. This kind of attack does* control-not* gate twice on two photons to steal one bit information and cause information leakage problem. Because our protocol uses* nonlocal swap* gate to exchange users' message, no secret information is transmitted during photons distribution, so our protocol can resist this attack.

*(4) The Forcible Measurement Attack *[34]. This attack measures photons during transmission to get secret message, but like the former attack, in this proposed protocol, transmitted photons are without carrying message, so this attack is invalid to our protocol.

*Participant Attack.* We focus on two sources of participant attack: attacks from the controller and attacks from the legitimate users. First, we discuss how the controller might steal the secret message, and the situation in which one of the legitimate users is dishonest.

**Figure 11:**
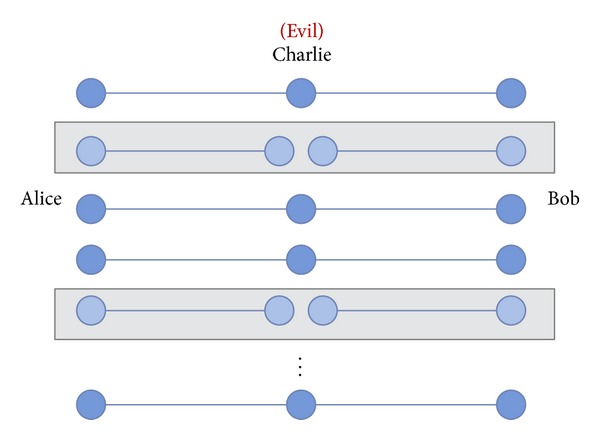
The scenario of participant attack in which Charlie (controller) inserts his EPR pairs to replace GHZ states.

**Figure 12:**
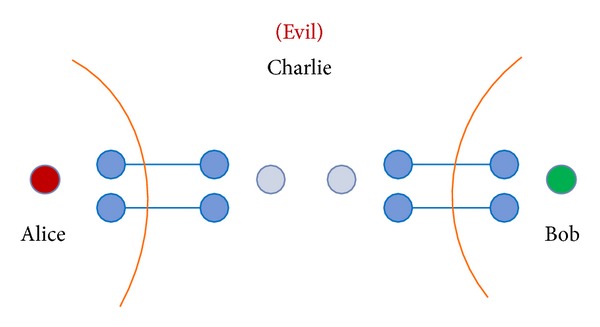
The scenario of participant attack 1 in which Charlie (controller) steals the secret messages by the* nonlocal swap* gate scheme.

*(1) The Man in the Middle Attack by Charlie.* We suppose that Charlie prepares some additional EPR pairs with the intent of stealing the secret messages by the* nonlocal swap* gate scheme [see Figure 12]. Before Charlie sends the sequence of *A* particles and *B* particles to Alice and Bob, he inserts 2*k*_*c*_ EPR pairs to replace *k*_*c*_ GHZ states [see Figure 11]. After this, Charlie sends *A*′ sequence and *B*′ sequence (*A*′ and *B*′ sequences represent the sequences that contain Charlie's attack EPR pairs) to Alice and Bob. If Charlie is not detected, and his EPR pairs are used as the quantum resources for the legitimate users to exchange their secret message, he can obtain the secret message from Alice and Bob. Since the quantum resources are kept between the legitimate users and the controller, he can mimic Alice and Bob's actions in order to obtain the secret message.

**Figure 13:**
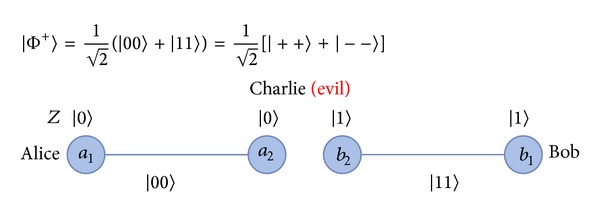
The scenario of participant attack 1 in which Charlie (controller) steals the secret messages by the* nonlocal swap* gate scheme.

However, the evil Charlie would be found out in the quantum channel. Let us show you the error detection rate that the legitimate users find the errors in the quantum channel and calculate the evil Charlie's successful rate. After Alice and Bob confirm with the evil Charlie that they have received all the sequences of particles *A*′ and *B*′ (*A*′ and *B*′ sequences are represented in a sequence that has contain Charlie's EPR pairs, respectively, they have an order to choose the random enough subset of *A* and *B* sequence for checking quantum channel security. Assume that the evil Charlie inserts the 2*k*_*c*_ EPR pairs, then the legitimate users have *k*_*c*_/*N* probability (Charlie prepares a group of *N* three-particles GHZ states.) to choose the evil Charlie's EPR pairs. If one of the legitimate users chooses the particle that is one of the EPR pairs from the evil Charlie for the channel check, the legitimate users have 1/2 probability to find the error. Assume that Alice chooses one of the *A*′ particles that is one of the EPR pairs $\left(1/\sqrt{2}\right){\left(|00\rangle +|11\rangle \right)}_{a_{1}a_{2}}$ and the corresponding particle in *B*′ sequence that is EPR pair $\left(1/\sqrt{2}\right){\left(|00\rangle +|11\rangle \right)}_{b_{1}b_{2}}$. Here, Alice keeps the qubit *a*_1_ and Bob keeps the qubit *b*_1_, and then the evil Charlie keeps the qubits *a*_2_ and *b*_2_ [see Figure 13].

However, Charlie would be found out in the quantum channel. The following shows the error detection rate of the legitimate users finding the errors in the quantum channel, and the calculation of Charlie's attack success rate. After Alice and Bob confirm with Charlie that they have received all the sequences of particles *A*′ and *B*′, respectively, they have an order to choose the sufficiently random subset of *A* and *B* sequence for checking the quantum channel security. Assume that Charlie inserts the 2*k*_*c*_ EPR pairs; then, the legitimate users have a *k*_*c*_/*N* probability (Charlie prepares a group of *N* three-particle GHZ states) of choosing Charlie's EPR pairs. If one of the legitimate users chooses the particle that is one of the EPR pairs from Charlie for the channel check, the legitimate users have a 1/2 probability of finding the error. Assume that Alice chooses one of the *A*′ particles that is one of the EPR pairs $\left(1/\sqrt{2}\right){\left(|00\rangle +|11\rangle \right)}_{a_{1}a_{2}}$ and the corresponding particle in *B*′ sequence that is EPR pair $\left(1/\sqrt{2}\right){\left(|00\rangle +|11\rangle \right)}_{b_{1}b_{2}}$. Here, Alice keeps the qubit *a*_1_ and Bob keeps the qubit *b*_1_, and then Charlie keeps the qubits *a*_2_ and *b*_2_ [see Figure 13].

If Alice (Bob) measures the qubit *a*_1_ (*b*_1_) using* Z*-basis, the measurement result will have a 1/2 probability of collapsing into |00〉_*a*_1_*a*_2__ (|00〉_*b*_1_*b*_2__) or |11〉_*a*_1_*a*_2__ (|11〉_*b*_1_*b*_2__. There are two choices for Charlie to publish his quantum state. If the two EPR pairs share the same measurement results, Charlie will not be found out. Conversely, Charlie has a 1/2 probability of failure. In other words, if Alice (Bob) measures the qubit *a*_1_ (*b*_1_) using* X*-basis, the measurement result will have a 1/2 probability of collapsing into |++〉_*a*_1_*a*_2__ (|++〉_*b*_1_*b*_2__) or |−−〉_*a*_1_*a*_2__ (|−−〉_*b*_1_*b*_2__). When the qubits *a*_1_ and *b*_1_ are |+〉 or |1〉, Charlie has a 1/2 probability of not being found out.

The overview of participant attack 1: Charlie prepares *N* GHZ states that include 2*k*_*c*_ EPR pairs inserted into the *A* and *B* sequences for detecting eavesdroppers and quantum resources. The legitimate users randomly choose *m* GHZ states together for detecting quantum channels. Therefore, when the legitimate users choose one of *N* GHZ states, Charlie has a ((*k*_*c*_/*N*) × (1/2) + ((*N* − *k*_*c*_)/*N*) × 100%) probability of not being found in the quantum channel. However, if the legitimate users choose *m* number of the *N* GHZ states for detecting quantum channels, the error detection rate is 1 − ((*k*_*c*_/*N*) × (1/2) + ((*N* − *k*_*c*_)/*N*) × 100%)^*m*^ for the legitimate users and controller; Charlie is found in the quantum channel regardless of whether the measuring basis is* X*-basis or* Z*-basis.

**Figure 14:**
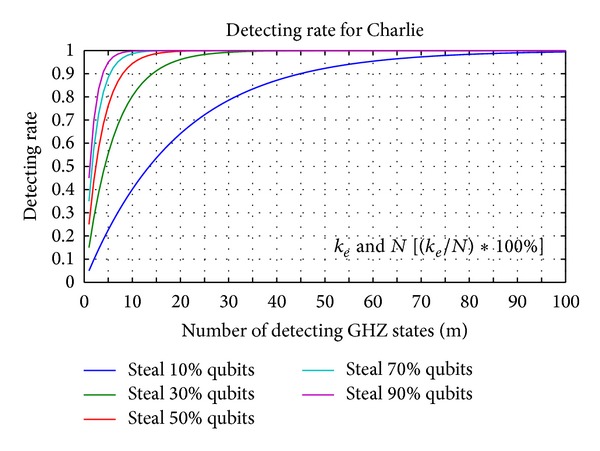
The different percentages of *k*_*c*_ in *N* GHZ states corresponding to the error detection rate and the number of detecting GHZ states. (*k*_*c*_: the number of GHZ states replaced by Charlie's EPR pairs. *N*: the number of GHZ states which are prepared from Charlie).

**Figure 15:**
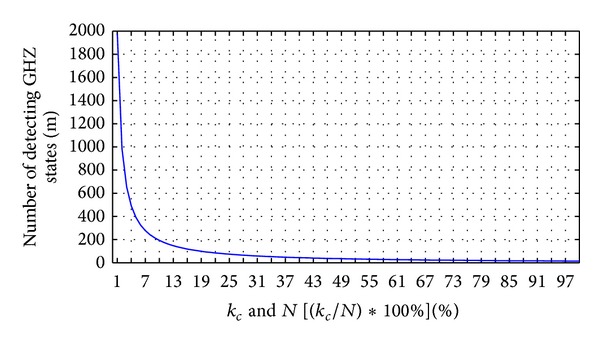
The legitimate users ensure quantum channel security with 100% probability corresponding to the number of detecting GHZ states and percentage of replaced GHZ states.

Figures 14 and 15 display the relation between the three parameters *m*, *k*_*c*_, and *N*. In Figure 14, we display five percentages of *k*_*c*_ in *N* GHZ states corresponding to the error detection rate and the number of detecting GHZ states. The legitimate users can depend on the error detection rate to decide how many *m* GHZ states must be used to ensure the quantum channel security. For example, suppose that Charlie prepares 100 GHZ states that include 100 EPR pairs to replace 50 GHZ states for the legitimate users. According to the line of 50% *N* in Figure 14, the legitimate users choose 17 GHZ states for checking the quantum channel, finding the error in the quantum channel with 99.2483% probability. When the legitimate users increase the number of GHZ states for checking the quantum channel to 51, there is a 100% probability of the legitimate users finding the error in the quantum channel. Therefore, the higher the number of GHZ states replaced, the fewer detecting GHZ states are required by the legitimate users to find the error in the quantum channel.

Figure 15 illustrates that the legitimate users ensure the quantum channel security with 100% probability corresponding to the number of detecting GHZ states and percentage of the replaced GHZ states. As with Figure 15, the higher the number of GHZ states replaced, the fewer detecting GHZ states required by the legitimate users to find the eavesdropper. Conversely, the legitimate users need to consume more detecting GHZ states to detect the quantum channels when Charlie replaces fewer GHZ states to be EPR pairs.

*(2) Dishonest Condition between Legitimate Users.* Some QSDC protocols may exhibit conditions that allow one of the legitimate users to derive the quantum information or secret message from the other one first, without assisting the other one in decoding the quantum information or secret message. The dishonest user may publish an incorrect measurement result, giving the other one an incorrect secret message, while they themselves obtain the correct secret message.

In our protocol, only the controller knows the initial GHZ state and EPR pairs, so the legitimate users are unable to know how to use the unitary operation to transfer their qubit state correctly. In addition, neither user has priority in obtaining the secret message in our protocol, as both receive the secret message from the other simultaneously.

Moreover, if one of the legitimate users deliberately announces an incorrect result to the controller, the controller will consequently give both legitimate users an erroneous unitary operation to transfer their qubit states, resulting in both users simultaneously receiving erroneous quantum information or secret messages. Assume that the measurement results of qubits 1, 2, 4, and 5 are |0〉_1_, |0〉_2_, |1〉_4_, and |1〉_5_, Charlie depends on their measurement result and his qubit 3 result |0〉_3_ to deduce that Alice and Bob need to apply the *Z* gate to transfer their qubit 0 (*c* | 0〉_0_ − *d* | 1〉_0_) and qubit 6 (*a* | 0〉_6_ − *b* | 1〉_6_). Charlie will announce the unitary operation (*Z* gate) for the legitimate users to transfer their qubits 0 and 6 as the correct results (*c* | 0〉_0_ + *d* | 1〉_0_) and (*a* | 0〉_6_ + *b* | 1〉_6_) that the legitimate users want to send to each other as follows:

(15) H134C46C02C65C10(a|0〉+b|1〉)0 ⊗12(|000〉+|111〉)134⊗(c|0〉+d|1〉)6⟶|01〉25 ⊗122[|000〉134(c|0〉+d|1〉)0(a|0〉+b|1〉)6+|001〉134(c|0〉−d|1〉)0(a|0〉−b|1〉)6+|010〉134(c|0〉−d|1〉)0(a|0〉−b|1〉)6+|011〉134(c|0〉+d|1〉)0(a|0〉+b|1〉)6+|100〉134(c|0〉−d|1〉)0(a|0〉−b|1〉)6+|101〉134(c|0〉+d|1〉)0(a|0〉+b|1〉)6+|110〉134(c|0〉+d|1〉)0(a|0〉+b|1〉)6+|111〉134(c|0〉−d|1〉)0(a|0〉−b|1〉)6].

**Figure 16:**
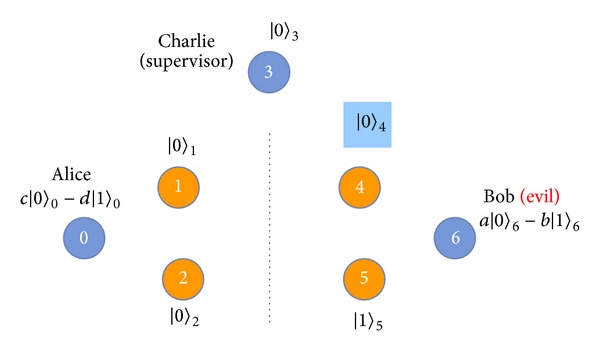
Bob publishes an incorrect measurement result of qubit 4.

However, a situation may arise in which one of the legitimate users publishes an incorrect measurement result and lets the other gain the wrong secret message, while themselves obtaining the correct secret message. Assume Bob is dishonest and deliberately publishes the wrong measurement result of qubit 4 |0〉_4_ for Alice and Charlie [see Figure 16]. Charlie depends on the incorrect measurement result to tell the legitimate users to apply the wrong gate (*I* gate) to transfer their qubits. The result is that Alice cannot receive the correct secret message from Bob, while Bob hopes to receive the correct secret message from Alice.

However, can Bob depend on the measurement results of qubits 1, 2, 4, and 5 to deduce what unitary operation he needs to perform on qubit 6 to obtain Alice's secret message successfully? The answer is no; only Charlie knows the initial state of quantum resources, so only Charlie knows the unitary operation to transfer the legitimate users' qubits 0 and 6. The unitary operations are not only *I* and *Z* gates, but also *X* and *Y* gates. The unitary operations follow different quantum resources (a GHZ state and an EPR pair) and have different applications. Even if Bob knows the unitary operation of only *I* and *Z* gates, and the measurement results of qubits 1, 2, 4, and 5, he still does not know the measurement result of Charlie's qubit 3. Bob has a 1/2 probability of correctly guessing that the measurement result of qubit 3 is |0〉_3_ or |1〉_3_. This means that Bob has a 1/2 probability of guessing the correct unitary operation gate by himself to obtain Alice's secret message. In other words, if Bob repeats the action *i* times, his failure rate would be 1 − (1/2)^*i*^. Therefore, according to the game theory presented by Nash Jr. [35], in order for the legitimate users to obtain the quantum information from each other, being honest to each other serves them best.

Our protocol not only defends against external attack (man in the middle attack), but also protects against legitimate users lying to the controller and guards against the controller stealing the secret message from the legitimate users by himself. Furthermore, with our protocol employed, since there is no transmission between the legitimate users, Eve has no opportunity to steal the secret message from the quantum channel.

## 5. Performance Comparison

We analyse the performance of the four protocols: Gao2005 [36], Dong2011 [37], Man2006 [38], and Dong2008 [39] and compare it with our protocol. There are two controlled one direction QSDC protocols and two controlled bidirectional QSDC protocols to be compared with our protocol. We briefly introduce these protocols and our protocol below.

Gao2005 is a controlled one direction QSDC scheme using GHZ state and teleportation. This protocol requires a GHZ-like state (three entangled qubits) to transmit quantum information and classical messages. In addition, Charlie publishes his result by one classical bit, and Alice announces her result by two classical bits, so the cost of Gao2005 is 3 qubits and 3 classical bits for one direction work. Here, the classical bits are used to communicate with each other in the classical channel. If the users want to exchange messages in Gao2005, they need to perform the protocol twice, so the cost is multiplied by two, consisting of 6 qubits and 6 classical bits. However, in Gao2005, the legitimate users receive the secret messages in order, rather than simultaneously, and this protocol cannot protect against the dishonesty of one user (participant attack (2)). Dong2011 presented a controlled one direction QSDC based on teleportation similar to Gao2005 above. The cost and security of Dong2011 are the same as those of Gao2005. The only difference between Gao2005 and Dong2011 is the type of secret message. Moreover, Gao2005 can transmit any unknown qubits, but Dong2011 can change the type of the secret message to pure states. Dong2011 is no more flexible than Gao2005 in transmitting legitimate users' secret messages. Hence, the contribution of Dong2011 is dubious.

Our protocol is a controlled bidirectional QSDC protocol with a GHZ state and an EPR pair. The legitimate users need to publish their respective measurement results by two classical bits, and the controller needs to tell the legitimate users how to transfer their qubit by two classical bits. The cost of our protocol is 5 qubits and 5 classical bits. The legitimate users receive the secret messages from each other simultaneously, and they can transmit any unknown quantum bit to each other. The security of our protocol is more reliable than that of the above protocols because there are no transmitted qubits carrying the secret messages between the legitimate users and the controller. Our protocol not only protects against external attack, but also prevents one legitimate user from being dishonest to the other. Furthermore, Collins et al. [23] note that the apparatus implementing the swap gate must use two EPR pairs as an internal nonlocal resource. Based on the* nonlocal swap* gate, the minimal quantum resource is 4 qubits. Our protocol, however, is a controlled bidirectional QSDC protocol that needs to add one qubit for the controller to control it. Therefore, our protocol has a minimal quantum resource cost (5 qubits) that can exchange any unknown qubit to each other.

According to Table 1, the cost of our protocol is one less qubit than that of Gao2005, because Gao's protocol uses one GHZ-like state (3 qubits) for work and 3 classical bits for public results at a time. In order to compare our protocol with the controlled bidirectional QSDC protocol, we have to work twice with the CQSDC protocol. The CQSDC protocols and our protocol can all transmit the quantum bits and classical bits to each other, but in terms of security, Gao2005 and Dong2011 are vulnerable to participant attack 2 between legitimate users, and they cannot transmit secret messages simultaneously.

Next, we choose two controlled bidirectional QSDC protocols, Man2006 and Dong2008, for comparison with our protocol. Man2006 shares a GHZ state for a controller and two legitimate users. If the legitimate users want to exchange their secret messages, they perform the unitary operation (one unitary operation can be represented by two classical bits) on their qubit and send it back to the controller. The controller will publish his GHZ measurement result to allow the two legitimate users to decode the secret messages from each other. Finally, the cost of Man2006 is three qubits and three classical bits for one time. However, Man2006 cannot transmit quantum information, and is vulnerable to participant attack 2. Because the secret message is made up of classical bits, the cost of the secret message might be lower than the quantum resources in Man2006. In terms of security of Man2006, it is vulnerable to attack by eavesdroppers stealing the qubits carrying the secret message in the transmissions between the legitimate users and the controller. Dong2008 is a controlled bidirectional QSDC protocol, in which legitimate users exchange their secret messages using entanglement swapping with two GHZ states. The controller first measures his two particles and publishes their measurement results by 2 classical bits. The legitimate users then need to Bell-measure their two particles and publish their Bell-measurement results by 2 classical bits, respectively. The cost of Dong2008 is 6 quantum bits and 6 classical bits for the legitimate users to exchange their secret messages at a time. Even though there are no transmissions with qubits carrying secret messages in Dong2008, it is also vulnerable to participant attack 2. In addition, since Dong2008 only transmits classical bits, the cost of sending the secret messages may be lower than the quantum resources of Man2006.

Man2006, Dong2008, and our protocol are controlled bidirectional QSDC protocols. As shown in Table 1, the cost of our protocol's quantum resources is higher than that of Man2006, but Man2006 cannot transmit any unknown qubits. The users can exchange two classical bits at a time in Man2006 and Dong2008, which is one bit more than our protocol. However, classical bits are cheaper than qubits. Our protocol, therefore, is more efficient than the above protocols. Man2006 and Dong2008 are also vulnerable to participant attack 2. In summary, our protocol is more efficient than other protocols, and provides the security for the legitimate users to exchange their secret messages with minimal quantum resources.

## 6. Conclusion

In this paper, we proposed a controlled bidirectional quantum secure direct communication using a* nonlocal swap* gate to simultaneously exchange quantum information or classical messages without transmitting the qubits carrying the secret messages. The legitimate users must have permission from a controller to exchange their respective quantum information or secret messages. Our protocol not only protects against external attack, but also against participant attack. In addition, our protocol uses minimal quantum resources for legitimate users to transmit any unknown qubits in controlled bidirectional QSDC protocols. It is secure against eavesdropping attacks, and the controller has no access to the quantum information or secret messages in our protocol. Therefore, our design of a novel CBQSDC protocol based on a* nonlocal swap* gate is quite secure, reliable, and confidential.

## Figures and Tables

**Table 1:** Comparison of CQSDC and CBQSDC protocols with our protocol.

			Scheme		
	Gao2005	Dong2011	Man2006	Dong2008	Ours
Protocol type	CQSDC	CQSDC	CBQSDC	CBQSDC	CBQSDC
Resource cost of two directional transmission	6 Q and 6 C	6 Q and 6 C	6 Q and 6 C	6 Q and 6 C	5 Q and 6 C
Secret message type	C/Q	C/Q	C	C	C/Q
Received classical bits	1 C	1 C	2 C	2 C	1 C
Received quantum bits	1 Q	1 Q	0	0	1 Q
Controller	Yes	Yes	Yes	Yes	Yes
Classical message exchange	Yes	Yes	Yes	Yes	Yes
Quantum information exchange	Yes	Yes	No	No	Yes
No transmission	Yes	Yes	No	Yes	Yes
Honest condition between legitimate users	No	No	No	No	Yes

C: classical bits; Q: quantum bits.
